# Comparative of metagenomic and targeted next-generation sequencing in lower respiratory tract fungal infections

**DOI:** 10.3389/fcimb.2025.1534519

**Published:** 2025-05-26

**Authors:** Zhiyang Chen, Xin Liu, Li Tan, Xing Lyu, Qichen Long, Weimin Wu, Zhe Guo, Zhenni Liu, Ziyang Li, Min Hu

**Affiliations:** ^1^ Department of Clinical Laboratory, The Second Xiangya Hospital of Central South University, Changsha, China; ^2^ Molecular Diagnostic Technology Hunan Engineering Research Center, Changsha, China; ^3^ Clinical Medical Research Center for Molecular Diagnosis of Infectious Diseases in Hunan Province, Changsha, China

**Keywords:** mNGS, tNGS, diagnosis, invasive pulmonary fungal infection, fungal

## Abstract

**Objectives:**

This study aims to compare the diagnostic efficiency and consistency of metagenomic next-generation sequencing (mNGS) and targeted next-generation sequencing (tNGS) in patients with lower respiratory tract fungal infections.

**Methods:**

A total of 115 patients with probable pulmonary infection between September 2022 and April 2023 were enrolled at the Second Xiangya Hospital, Changsha, China, of which 61 were clinically diagnosed with invasive pulmonary fungal infection (IPFI) and 54 were non-IPFI cases. All patients received bronchoalveolar lavage, with mNGS, tNGS, and cultures being conducted paralleled. Diagnostic effectiveness and consistency in detecting microorganisms were compared.

**Results:**

Both mNGS and tNGS showed high sensitivity rates of 95.08% each, with specificity of 90.74% and 85.19%, respectively. They also demonstrated positive predictive values (PPVs) of 92.1% and 87.9% and negative predictive values (NPVs) of 94.2% and 93.9%, respectively, in diagnosing IPFI. The sensitivity and NPV of mNGS and tNGS were superior to that of any individual or combined conventional microbiological tests (CMTs) (P < 0.05). The consistency of culture with mNGS and tNGS was 48.70% and 50.43%, respectively. For fungal detection, *Pneumocystis jirovecii* (26/61, 42.6%; and 28/61, 45.9%), *Candida albicans* (19/61, 31.1%; and 21/61, 34.4%), and *Aspergillus fumigatus* (16/61, 26.2%; and 15/61, 24.6%) are most prevalent for mNGS and tNGS in enrolled cases, and the detection rate was greatly higher than that of culture. Furthermore, mNGS and tNGS were capable of diagnosing mixed infections in 65 and 55 out of the 115 cases, whereas only nine cases of bacterial-fungal infection were detected by culture.

**Conclusion:**

The diagnostic efficacy of mNGS and tNGS was comparable to that of identified IPFI. NGS-based methodologies present a promising tool for detecting IPFI, which can be a good supplement to CMT.

## 1 Background

Despite the utilization of existing antifungal drugs, invasive fungal diseases (IFDs) are estimated to result in approximately 6.5 million incidence and 3.8 million deaths worldwide annually (Denning, 2024). Among the IFDs, invasive pulmonary fungal infections (IPFIs) are frequently observed in clinical settings, notably among individuals suffering from lung cancer, diabetes, tuberculosis, compromised immune systems, and those on prolonged antibacterial medication. In recent years, the prevalence and mortality of IPFI are generally increasing (Parums, 2022). Hence, rapid identification of pathogens is of great importance for IPFI.

At present, the conventional techniques for diagnosing IPFI encompass smears, cultures, antigen-antibody assays, and molecular biology tests. However, these existing methods are challenging to meet clinical needs due to low positivity rates and because they are time-consuming and need prior assumptions. Patients who test negative by conventional methods are treated with empirical antibiotics, which may lead to potential reinfection and adverse reactions (Huang et al., 2020). In order to meet the current need for prompt identification of pathogens and initiate timely and appropriate treatment, next-generation sequencing (NGS) has seen significant advancements this year, which becomes a front-line diagnostic in identifying rare pathogens and in the assessment of patients who may be suffering from severe infections (Wilson et al., 2014; Miller et al., 2019; Zhu et al., 2020; Zheng et al., 2021).

The common applications of NGS in diagnostic laboratories include metagenomic NGS (mNGS) and targeted NGS (tNGS). mNGS allows for the comprehensive detection of a wide range of pathogens, including viruses, bacteria, fungi, and parasites. However, the simultaneous detection of both DNA and RNA processes is economically challenging (Li et al., 2022). tNGS based on multiplex PCR amplification or probe capture enriches nucleic acid of known pathogen (Zhao et al., 2021; Singh, 2022). As a more cost-effective and high sensitivity assay, tNGS is gaining attention in clinical infectious management (Chen et al., 2024). To date, there has been a lack of comparative analysis between mNGS and tNGS in IPFI. Therefore, we conducted this retrospective study among patients with IPFI to evaluate the diagnostic efficacy of mNGS, tNGS, and conventional microbiological tests (CMTs) to assess the practical application of mNGS and tNGS in the clinical management for managing invasive IPFIs.

## 2 Methods

### 2.1 Participants and study design

This retrospective case series involved a total of 115 patients with probable pulmonary infection between September 2022 and April 2023 in the Second Xiangya Hospital, Central South University, China. All patients received bronchoalveolar lavage, with mNGS, tNGS, and cultures being conducted paralleled.

The inclusion criteria for this study were as follows: (i) patients with clinical manifestations of pulmonary infections; (ii) underwent bronchoalveolar lavage; (iii) implementation of mNGS, tNGS, and CMTs; and (iv) availability of complete clinical data. The exclusion criteria included the following: (i) individuals lacking concurrent culture, mNGS, and tNGS tests; and (ii) cases with incomplete clinical medical records.

The diagnosis of IPFI or non-IPFI was based on a composite clinical judgement by a clinician team that consists of at least two senior clinicians/professors, including clinical symptoms (fever, respiratory symptoms, fungal-related radiological changes, etc.), microbiological evidence (G test, GM test, fungal staining, molecular methods, etc.), and host factors (the presence of immunosuppression). This study was approved by the Ethics Committee of the Second Xiangya Hospital, Central South University (LYF2022229). The study was considered exempt from informed consent as it was a retrospective observational cohort study.

### 2.2 Data collection

Demographic and clinical data were extracted from electronic medical records, including age; gender; underlying diseases; pulmonary imaging and laboratory findings (including Erythrocyte Sedimentation Rate (ESR), C-reactive Protein (CRP), Procalcitonin (PCT), and blood routine); results of CMTs, mNGS, and tNGS; and outcome.

### 2.3 mNGS sequencing and analysis

The methods of mNGS were the same as that described in our previously published article (Li et al., 2024). DNA was extracted from samples using the QIAamp^®^ UCP Pathogen DNA Kit, in which human DNA was removed. RNA extraction was performed using the QIAamp UCP pathogen mini kit, followed by Turbo DNase treatment to deplete the host DNA background. The RNA was then reverse-transcribed and amplified using the Ovation RNA-Seq system. After fragmentation, the library was constructed using the Ovation Ultralow System V2. Sequencing was carried out on the Illumina NextSeq 550 with single-end 75-bp reads. During data analysis, low-quality reads were removed using fastp. Human sequences were identified and excluded by aligning to the hg38 genome using the Burrows-Wheeler Aligner software. The remaining microbial reads were aligned to the database using Short Nucleotide Alignment Program (SNAP) to identify pathogens. Regarding the cutoff values, for pathogens with background reads in the negative control, a given species or genus was reported as a positive detection if the reads-per-million (RPM) ratio was ≥10. The RPM ratio was calculated as the RPM of the sample divided by the RPM of the no-template control (RPMsample/RPMNTC). For pathogens without background reads in the negative control, the RPM thresholds for positive detection were set as follows: for bacteria, mycoplasma, chlamydia, DNA viruses, and fungi, ≥3 reads; for the *Mycobacterium tuberculosis* complex, ≥1 read. Finally, clinical symptoms, laboratory findings, and the immune status of patients will be taken into account to comprehensively assess whether the detected microorganisms are potential pathogens.

### 2.4 tNGS sequencing and analysis

#### 2.4.1 Sample preparation

A volume of 650 μL of the sample was liquefied by combining it with an equal volume of dithiothreitol (80 mmol/L) in a 1.5-mL centrifuge tube. The sample used was from the same tube of bronchoalveolar lavage fluid (BALF) collected from the patient, and all samples were stored at −20°C and processed within 24 h after collection. The mixture was homogenized for 15 s using a vortex mixer. Meanwhile, a positive control and a negative control from the Respiratory Pathogen Detection Kit (KS608-100HXD96, KingCreate, Guangzhou, China) were set up to monitor the whole experiment process of tNGS.

#### 2.4.2 Nucleic acid extraction

Five hundred microliters of the homogenate was utilized for total nucleic acid extraction and purification via the MagPure Pathogen DNA/RNA Kit, following the manufacturer’s protocol.

#### 2.4.3 Library construction and sequencing

We used the Respiratory Pathogen Detection Kit to construct the library. Two rounds of PCR amplification were performed with 198 pathogen-specific primers for ultra-multiplex PCR to enrich target sequences of bacteria, viruses, fungi, mycoplasma, and chlamydia (**Supplementary Table 1**). After amplification, PCR products were bead-purified and then amplified with primers having sequencing adapters and unique barcodes. The Qsep100 Bio-Fragment Analyzer (Bioptic, Taiwan, China) and Qubit 4.0 fluorometer (Thermo Scientific, Massachusetts, USA) were used to evaluate library quality and quantity, respectively. Library fragments were about 250–350 bp in size, and the library concentration was at least 0.5 ng/µL. The mixed library’s concentration was reevaluated and diluted to 1 nmol/L. Specifically, we use the following conversion formula: $\text{nmol}/\text{L}=\frac{\text{ng}/\text{μL}\times 10^{6}}{\text{L}\times 660\text{g}/\text{mol}}$ (660 g/mol, which represents the average molecular weight of double-stranded DNA; L denotes the fragment length, and, for this purpose, we consider 300 bp). We dilute the final library to 1 nmol/L with nuclease-free water. Then, 5 µL of the library is mixed with 5 µL of fresh 0.1 mol/L NaOH, vortexed, centrifuged, and incubated at room temperature for 5 min. The diluted and denatured library is sequenced on an Illumina MiniSeq using a KingCreate (Guangzhou, China) universal sequencing kit (KS107-CXR). Each library generates about 0.1 million single-end 100-bp reads on average.

#### 2.4.4 Bioinformatics analysis

Sequencing data were analyzed using the data management and analysis system (v3.7.2, KingCreate). The raw data underwent initial identification via the adapter. Reads with single-end lengths exceeding 50 bp were retained, followed by low-quality filtering to retain reads with Q30 > 75%, ensuring high-quality data. The single-ended aligned reads were then compared using the Self-Building clinical pathogen database to determine the read count of specific amplification targets in each sample. The reference sequences used for read mapping were database-curated from different sources, including GenBank database, RefSeq database, and Nucleotide database from National Center for Biotechnology Information.

#### 2.4.5 Report results’ output

According to the principle of targeted amplification of microbial sequences using specific primers, the amplicon coverage and normalized read count of detected pathogens were served as the primary result interpretation indicator. The following criteria were established to classify a microorganism as a potential pathogen: (i) bacteria (excluding *Mycobacterium tuberculosis* complex), fungi, and atypical pathogen: amplicon coverage ≥50% and normalized read count ≥50; (ii) viruses: amplicon coverage ≥50% and normalized read count ≥30; and (iii) *Mycobacterium tuberculosis* complex: normalized read count ≥10. Then, the patient’s medical history, symptoms, the immune status, and other laboratory results were assessed to conduct a comprehensive assessment by two experienced clinicians or clinical microbiologist independently. Conflicting interpretations were consulted with a senior physician to achieve a consensus.

### 2.5 Statistical analyses

Quantitative variables were represented as medians with accompanying ranges, and categorical variables were presented as counts with percentages. Wilson’s method was used to calculate 95% confidence intervals (CIs) for these proportions. The 2 × 2 contingency tables were established to determine sensitivity, specificity, positive predictive value (PPV), and negative predictive value (NPV). The McNemar’s test was used for comparisons of the diagnostic performance of CMTs, mNGS, and tNGS. The SPSS 27.0.1 software was used for data analysis, and *P* < 0.05 was considered statistically significant.

## 3 Results

### 3.1 Clinical characteristics

**Figure 1:**
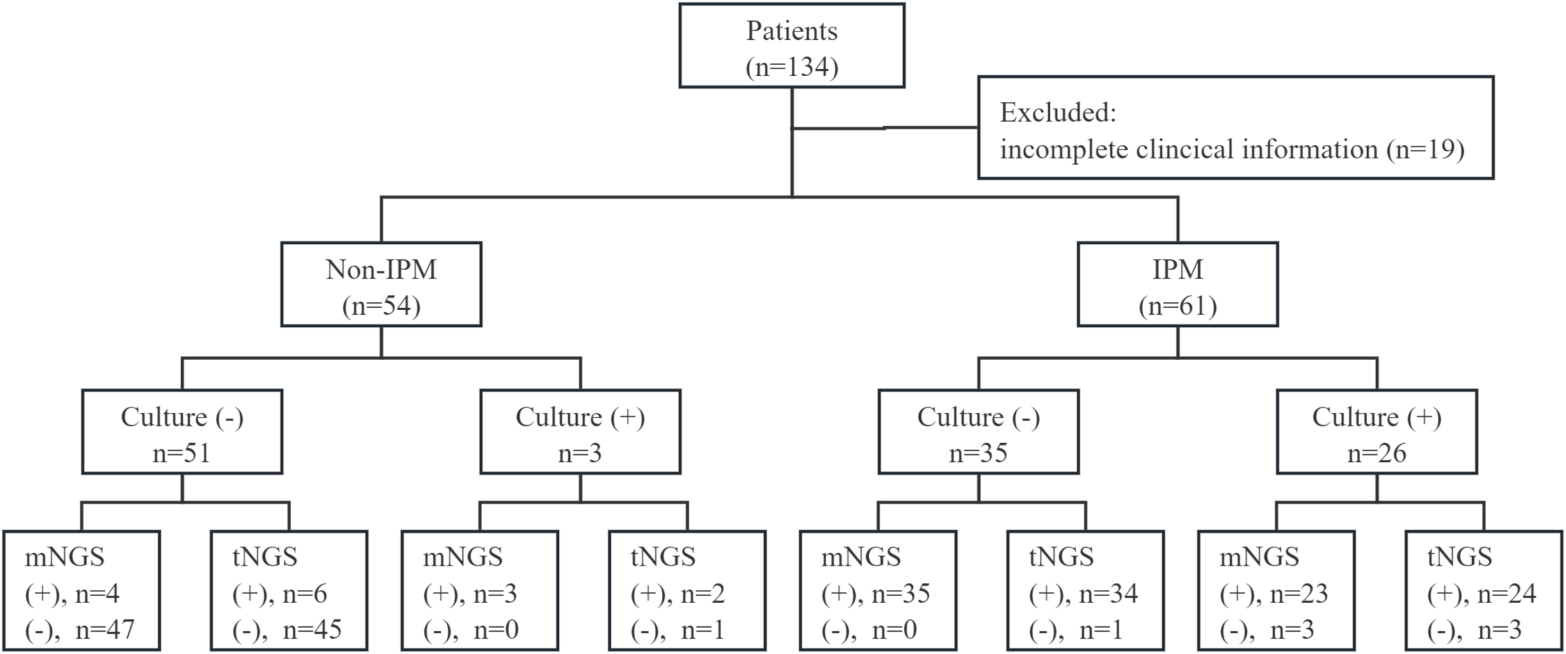
Overview of the research scheme and results of culture, mNGS, and tNGS. (+), positive; (−), negative.

As shown in **Figure 1**, a total of 134 patients with suspected lower respiratory tract infections, who underwent both BALF mNGS and tNGS, were enrolled. Of these, 19 patients had incomplete clinical information, and 115 patients were subjected to final analysis. Based on clinical diagnosis, patients were divided into non-IPFI (n = 54) and IPFI group (n = 61).

The clinical features of these two groups were analyzed and summarized in **Table 1**. The distribution of genders was comparable between the groups, yet the median age of the IPFI group was significantly higher than that of the non-IPFI group (*P* = 0.027). For laboratory findings, levels of C-reactive protein (*P* = 0.027), white blood cell count (*P =* 0.043), neutrophil percentage (*P* = 0.001), and the neutrophil-to-lymphocyte ratio (*P* = 0.001) were all markedly elevated in the IPFI group compared to those in the non-IPFI group. The most prevalent underlying conditions were malignancy in the non-IPFI group (35.2%) and hypertension in the IPFI group (37.7%). Regarding the typical radiological features on chest CT images, exudation was the most frequent feature in patients from both groups (*P* = 0.35). Nodules (*P* < 0.01), lymphadenopathy (*P* < 0.01), and masses (*P* < 0.01) were significantly more frequent in the non-IPFI group. In addition, ground-glass opacity was more prevalent in the IPFI group (*P* = 0.025).

**Table 1:** Demographics and clinical characteristics of the study cohort.

Characteristic	Non-IPFI group (n = 54)	IPFI group (n = 61)	*P-*value
Age, years, median (IQR)	57.22 ± 15.44	63.8 ± 15.88	0.027
Gender, male, N (%)	42 (77.8)	46 (75.4)	0.766
Laboratory findings			
ESR	56.66 ± 35.12	47.4 ± 29.65	0.174
CRP, median (IQR)	38.75 (8.79,79.80)	73.56 (20.3, 136.9)	0.027
PCT, median (IQR)	0.22 (0.07,0.84)	0.47 (0.085, 2.255)	0.123
WBC, median (IQR)	6.955 (5.805, 10.68)	9.62 (6.255, 13.61)	0.043
N%	74.667 ± 16.43	83.636 ± 13.04	0.001
L%, median (IQR)	13.70 (5.725, 24.6)	6.80 (3.25, 12.80)	0.001
NLR, median (IQR)	5.50 (2.565, 14.53)	13.13 (6.21, 28.89)	0.001
Comorbidities, N (%)			
Malignancy	19 (35.2)	14 (23.0)	0.148
Diabetes	9 (16.7)	19 (31.1)	0.071
hypertension	13 (24.1)	23 (37.7)	0.116
Chronic liver disease	9 (16.7)	11 (18.0)	0.847
Cardiovascular disease	12 (22.2)	17 (27.9)	0.486
Chronic kidney disease	10 (18.5)	20 (32.8)	0.082
Immune disease	4 (7.4)	5 (8.2)	0.875
Hemopathy	4 (7.4)	1 (1.6)	0.13
CT images, N (%)			
Exudation	33 (61.1)	32 (52.5)	0.35
Nodules	19 (35.2)	6 (9.8)	0.001
Lymphadenectasis	10 (18.5)	2 (3.3)	0.008
Mass	6 (11.1)	0 (0.0)	0.007
Cavitation	2 (3.7)	2 (3.3)	0.901
Ground-glass opacity	3 (5.6)	12 (19.7)	0.025
LOS, days, median (IQR)	17.00 (9.00, 25.25)	16.00 (10.00, 23.50)	0.638
Outcome, Death, N (%)	1 (1.9)	8 (13.1)	0.025

### 3.2 Diagnostic efficacy of mNGS, tNGS, and CMT methods

As shown in **Table 2**, on the basis of clinical comprehensive diagnosis, mNGS identified IPFI in 58 out of the 61 patients in the IPFI group and 5 out of 54 in the non-IPFI group, showing a sensitivity of 95.08%, specificity of 90.74%, PPV of 92.1%, and NPV of 94.2%; whereas tNGS showed sensitivity of 95.08, specificity of 85.19%, PPV of 87.9%, and NPV of 93.9%. The diagnostic efficacy of mNGS and tNGS was comparable (*P* > 0.05). As for CMTs, the sensitivity of culture, G test, and immunofluorescence (IF) was 42.62%, 37.84%, and 57.14%, respectively, with the specificity of 94.44%, 80.56%, and 89.47%. The combination use of the three CMTs identified only 36 of the 61 patients with IPFI, yielding sensitivity of 59.02%, specificity of 83.33%, PPV of 80%, and NPV of 64.3%. Both mNGS and tNGS demonstrated higher sensitivity and NPV compared to either individual or combined CMTs (*P* < 0.05). Additionally, the combination of CMTs with mNGS achieved the highest sensitivity at 100%, followed by the combination of CMTs with tNGS with sensitivity of 98.36%.

**Table 2:** Diagnostic performance of CMTs, mNGS, and tNGS for invasive pulmonary fungal infection.

Method	Result	Non-IPFI	IPFI	Sensitivity % (95% CI)	Specificity % (95% CI)	PPV % (95% CI)	NPV % (95% CI)
Culture	POS	3	26	42.62 (30.0–55.9)^abcd^	94.44 (84.6–98.8)^cd^	89.7 (73.5–96.4)	59.3 (53.8–64.6)^abcd^
	NEG	51	35				
G test	POS	7	14	37.84 (22.5–55.2)^abcd^	80.56 (64.0–91.8)	66.7 (47.8–81.4)^ab^	55.8 (48.3–62.9)^abcd^
	NEG	29	23				
IF	POS	2	20	57.14 (39.4–73.7)^abcd^	89.47 (66.9–98.7)	90.9 (72.3–97.5)	53.1 (42.9–63.1)^abcd^
	NEG	17	15				
CMTs	POS	9	36	59.02 (45.7–71.4)^abcd^	83.33 (70.7–92.1)	80.0 (68.0–88.3)	64.3 (56.6–71.3)^abcd^
	NEG	45	25				
mNGS	POS	5	58	95.08 (86.3–99.0)	90.74 (79.7–96.9)	92.1 (83.4–96.4)	94.2 (84.4–98.0)
	NEG	49	3				
tNGS	POS	8	58	95.08 (86.3–99.0)	85.19 (72.9–93.4)	87.9 (79.2–93.2)	93.9 (83.5–97.9)
	NEG	46	3				
CMTs + mNGS	POS	12	61	100.00 (94.1–100.0)	77.78 (64.4–88.0)	83.6 (75.5–89.3)	100
	NEG	42	0				
CMTs + tNGS	POS	14	60	98.36 (91.2–100.0)	74.07 (60.3–85.0)	81.1 (73.2–87.1)	97.6 (85.1–99.6)
	NEG	40	1				

a The difference was significant with mNGS based on the Chi-square test (*P* < 0.05).

b The difference was significant with tNGS based on the Chi-square test (*P* < 0.05).

c The difference was significant with CMTs + mNGS based on the Chi-square test (*P* < 0.05).

d The difference was significant with CMTs + tNGS based on the Chi-square test (*P* < 0.05).

### 3.3 Fungal detection by culture

**Figure 2:**
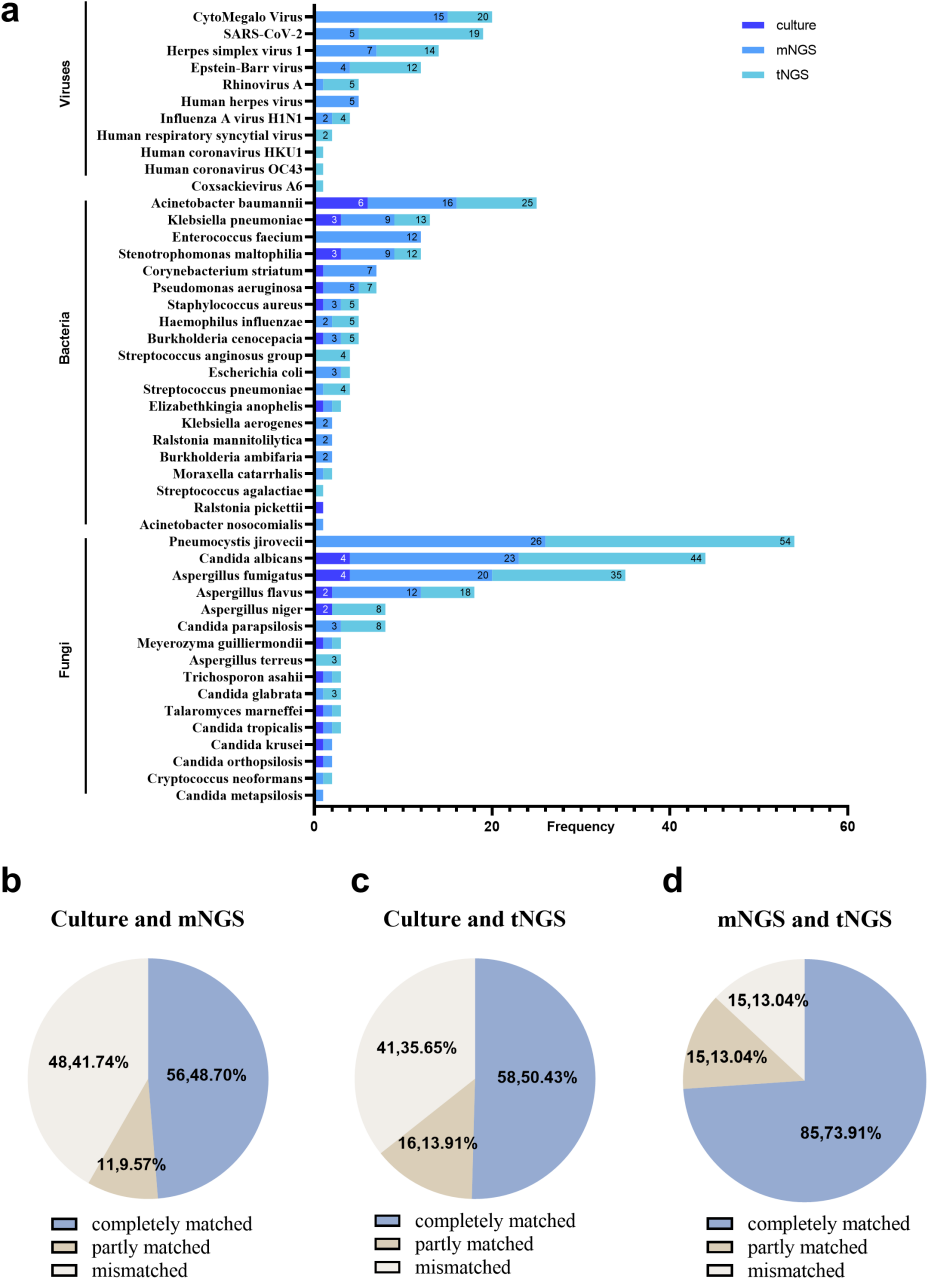
The pathogens distribution and the comparison of culture, mNGS, and tNGS in all cases. **(a)** Distribution of pathogens detected by culture, mNGS, and tNGS. **(b)** Culture and mNGS consistency. **(c)** Culture and tNGS results’ consistency. **(d)** mNGS and tNGS results’ consistency.

Among the 115 enrolled cases, culture, mNGS, and tNGS observed 19, 41, and 38 potential pathogens, respectively. The overall detection distribution of pathogens is shown in **Figure 2a**. The sensitivity for all pathogens and fungal pathogens using culture both varied significantly from mNGS and tNGS (*P* < 0.001), with culture of 46.09% and 24.35%, mNGS of 86.96% and 54.78%, and tNGS of 85.22% and 57.39%, respectively. In addition, mNGS and tNGS were comparable (*P* > 0.05).

In 61 patients with IPFI, fungal pathogens were isolated by culture in 26 cases. *Candida albicans* (15.38%) and *Aspergillus fumigatus* (15.38%) were the most frequently detected, followed by *Aspergillus niger* (7.69%) and *Aspergillus flavus* (7.69%). Out of the 26 cases, mNGS and tNGS had a complete match in identifying pathogens in 10 cases (38.46%). Moreover, mNGS and tNGS discovered additional fungal species in comparison to culture in 13 (50.00%) cases. Specifically, mNGS additionally detected *Pneumocystis jirovecii* (eight cases), *Candida parapsilosis* (two cases), *Candida albicans* (two cases), *Aspergillus flavus* (two cases), *Candida glabrata* (one case), and *Aspergillus fumigatus* (one case). On the other hand, tNGS uniquely identified *Pneumocystis jirovecii* (six cases), *Candida parapsilosis* (three cases), *Candida albicans* (three cases), *Candida glabrata* (two cases), *Aspergillus flavus* (two cases), *Aspergillus terreus* (one case), *Aspergillus niger* (one case), and *Aspergillus fumigatus* (one case). On the contrary, three cases that identified *Candida albicans* (one case), *Aspergillus fumigatus* (one case), and *Aspergillus niger* (one case) by culture were negative via mNGS. In addition, *Candida orthopsilosis* (one case) and *Candida krusei* (one case) were identified negative via tNGS. In the non-IPFI group, three cases of *Candida* were observed by culture and were consistent with mNGS and tNGS, combined with comprehensive clinical thinking, considering colonization.

### 3.4 Fungal detection by mNGS

Fifty eight of the 61 patients in the IPFI group tested positive for fungal detection by mNGS (95.08%). *Pneumocystis jirovecii* (42.62%) was the most frequently detected, followed by *Candida albicans* (31.15%) and *Aspergillus fumigatus* (26.23%). In all cases, the mNGS results were in complete agreement with the culture results in 56 (48.70%) cases, in partial agreement in 11 (9.57%) cases, and in complete disagreement in 48 (41.74%) cases (**Figure 2b**). In specific, in the IPFI group, the mNGS results were consistent with the culture results in 7 (12.07%) cases. In addition, the mNGS results were inconsistent with culture results in 41 (70.69%) cases (38 cases: mNGS was positive, whereas culture was negative, for fungal detection; and 3 cases: mNGS was negative, whereas culture was positive, for fungal detection). Among the 38 cases, mNGS detected fungal including *Pneumocystis jirovecii* (18 cases), *Candida albicans* (10 cases), *Aspergillus fumigatus* (seven cases), *Aspergillus flavus* (four cases), *Aspergillus niger* (two cases), *Cryptococcus neoformans* (one case), *Candida metapsilosis* (one case), and *Candida parapsilosis* (one cases). Five cases were identified fungal positive in the non-IPFI group, including a patient with lung cancer with *Pneumocystis jirovecii* (one case) and four cases of *Candida.*

### 3.5 Fungal detection by tNGS

Fifty eight of the 61 patients in the IPFI group tested positive for fungal detection by tNGS (95.08%). The top three detected pathogens of tNGS were consistent with those of mNGS; these are *Pneumocystis jirovecii* (45.90%), *Candida albicans* (34.43%), and *Aspergillus fumigatus* (24.59%). Compared with culture, in overall cases, the tNGS results were with a full consistency with the culture results in 58 (50.43%) cases, with partial consistency in 16 (13.91%) cases, and with complete inconsistency in 41 (35.65%) cases (**Figure 2c**). In the IPFI group, the tNGS results were in complete agreement with the culture results in 11 (18.03%) cases. Moreover, the tNGS results were in complete disagreement with culture in 35 (57.38%) cases (33 cases: tNGS was positive for fungal while culture was negative; and 2 cases: tNGS was negative, whereas culture was positive, for fungal detection). Among the 33 cases, tNGS detected fungal including *Pneumocystis jirovecii* (19 cases), *Candida albicans* (10 cases), *Aspergillus fumigatus* (four cases), *Aspergillus niger* (two cases), *Cryptococcus neoformans* (one case), *Aspergillus flavus* (one case), *Aspergillus terreus* (one case), and *Candida parapsilosis* (one case). Eight cases were identified fungal positive in the non-IPFI group, including a respiratory failure patient with one case of *Pneumocystis jirovecii* and seven cases of *Candida.*

For the comparison of mNGS and tNGS, the results in all cases were completely matched in 85 (73.91%) cases, partly matched in 15 (13.04%) cases, and mismatched in 15 (13.04%) cases (**Figure 2d**). Specifically, in mismatched cases, mNGS missed *Candida albicans* (two cases), *Aspergillus fumigatus* (one case), and *Pneumocystis jiroveciiwas* (one case) in four cases. Because low sequencing reads of colonized *Candida* was observed by mNGS, compared with the different reported rules between tNGS and mNGS, *Candida* was not reported by mNGS but tNGS in three cases. Compared with mNGS, tNGS missed *Aspergillus fumigatus* (two cases), *Pneumocystis jirovecii* (one case), and *Candida krusei* (one case). In addition, in other five cases, fungus was filtered because the detected sequence number was lower than the reported limit [*Pneumocystis jirovecii* (one case), *Aspergillus fumigatus* (one case), *Candida parapsilosis* (two cases), and *Aspergillus flavus* (one case)].

### 3.6 Detection of coinfections by culture, mNGS, and tNGS

In a total of 55 of the 115 patients (47.83%), coinfection was found using tNGS, increasing the detection rate of coinfection compared to that of culture (7.83%) (*P* < 0.001). It is worth noting that 10.43%, 13.04%, 11.30%, and 13.04% of patients were diagnosed as bacterial-fungal, bacterial-virus, fungal-virus, and bacterial-fungal-virus coinfections, respectively.

**Figure 3:**
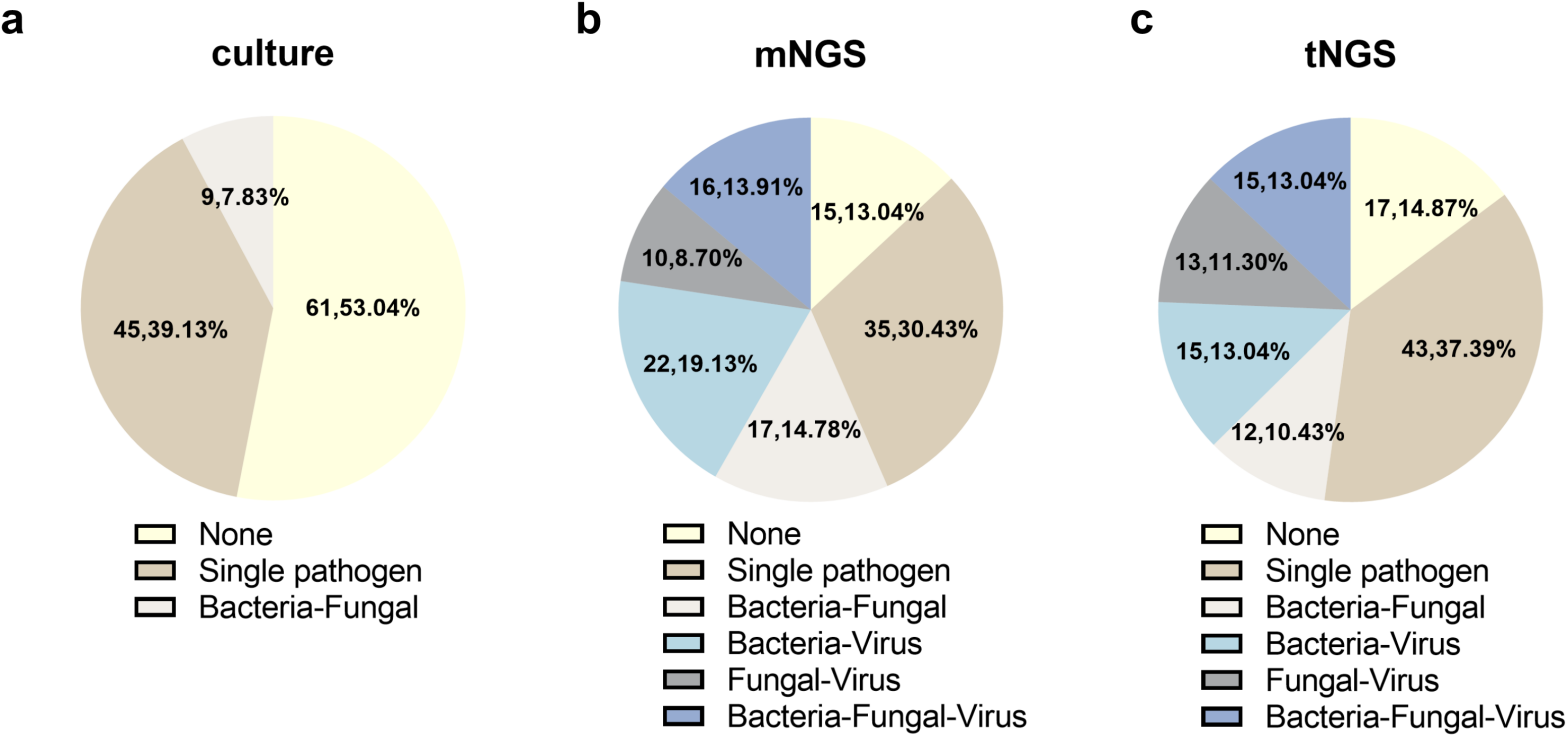
Coinfection detection of **(a)** culture, **(b)** mNGS, and **(c)** tNGS.

Sixty five out of the 115 patients (56.5%) were found coinfection using mNGS, which has no significant difference compared with tNGS. The detection rate of bacterial-fungal, bacterial-virus, fungal-virus, and bacterial-fungal-virus coinfections were 10.43%, 13.04%, 11.30%, and 13.04%, respectively (**Figure 3**).

## 4 Discussion

Patients presenting with IPFI often exhibit a range of underlying health conditions, posing a challenge in clinical management. The options for anti-fungal medications are limited, typically being both expensive and require prolonged treatment duration. Additionally, anti-fungal drugs often present significant adverse reactions and may be poorly tolerated by patients. Therefore, achieving an accurate diagnosis of IPFI is critically important.

The emergence of NGS has brought great progress to the diagnosis of infectious diseases. In recent years, mNGS has been widely accepted and used in clinical practice for early treatment or differential diagnosis of critically ill patients (Chiu and Miller, 2019; Greninger and Naccache, 2019; Chen et al., 2020). Our previous study showed that mNGS possesses greater sensitivity and a higher rate of clinical acceptance compared to culture when used as a reference for clinical diagnosis (Li et al., 2024), which was widely reported and supported by several studies. However, mNGS still has some limitations: easily influenced by human genes, expensive, and lack of standardization in experimental procedures and sequencing report interpretation. In contrast, tNGS involves the use of a panel of specific sequences from pre-selected pathogens, which offers higher specificity and cost-effectiveness and can eliminate the interference of human-derived genes (Wylie et al., 2015).

Upon reviewing relevant literature, it is found that there were few published studies compared the performance of tNGS and mNGS in lower respiratory tract infections (Huang et al., 2024; Sun L. et al., 2024; Wei et al., 2024). However, assessments of the clinical diagnostic value of tNGS in IPFI are limited. In the present study, two promising NGS-based detection methods, mNGS and tNGS, were compared in fungal diagnosis effectiveness in BALF. The tNGS panel used in this study was a primer or probe specifically constructed for 198 pathogens.

In our study, according to composite clinical judgement, 61 patients with IPFI and 54 non-IPFI cases were identified. We then compared the diagnostic efficiency and consistency between traditional culture method, mNGS, and tNGS, to assess the clinical validity of NGS in detecting fungi. In general, the diagnostic efficacy of mNGS and tNGS was comparable in identified IPFI without statistically different (*P* > 0.05), which aligns with the findings from previous studies (Li et al., 2022; Huang et al., 2024). Meanwhile, mNGS and tNGS, with their unbiased detection advantages, can identify pathogens that are difficult to detect by traditional culture methods, such as *Pneumocystis jirovecii*, *Cryptococcus neoformans*, viruses, and non-tuberculous mycobacteria. Our findings indicated that mNGS had equivalent sensitivity to tNGS, but with slightly higher specificity, PPV, and NPV compared to those of tNGS. However, tNGS identified more *A. terreus* complex, *A. niger* complex, and *C. parapsilosis* than mNGS. Moreover, in our study, the sensitivity and NPV of mNGS and tNGS were markedly higher than those of the combined CMTs, which include culture, G test, and IF, as well as traditional pathogen detection methods alone (*P* < 0.05). The combination of CMTs with mNGS identified all patients with IPFI with a NPV of 100%, whereas the combination of CMTs with tNGS missed one patient with IPFI (case 31). *A. fumigatus* was detected by mNGS (number of sequences: 6) in the 31st patient with IPFI with GM test being positive and with culture being negative. Interestingly, *P. jirovecii*, an opportunistic fungus often seen in immunocompromised patients with mixed infections, was the most frequently detected in both NGS-based assays in our study. This prevalence may be associated with the older age of the patients and the presence of underlying diseases. Coinfections with *P. jirovecii*, cytomegalovirus (CMV), and Herpesviruses such as Epstein-Barr virus (EBV) have been widely reported, and our findings are in line with previous studies (Rucar et al., 2024).

The detection efficiency of two-NGS based assays is excellent; however, there are also cases that are not consistent with the positive culture results. mNGS failed to detect fungi in three patients (cases 18, 28, and 37) of *C. albicans*, *A. fumigatus*, and *A. niger* complex, respectively, whereas culture and tNGS were positive. The three cases were confirmed as true positives by traditional culture and tNGS. However, the low sequence counts detected by tNGS, which barely met the reporting threshold, suggested low pathogen loads. Therefore, we suppose the false negative values of mNGS were likely due to these low pathogen loads. In addition, tNGS missed *C. orthopsilosis* and *P. kudriavzevii*, whereas culture and mNGS were positive in case 48 and case 59, respectively. These two cases were confirmed true positive as well. Since the differences have no pattern and are not caused by the differences in the detection ranges of mNGS and tNGS, these differences may be related to reasons such as degradation of samples due to storage or wet experimental performance.

Except for fungi, the detection performance of the two NGS-based assays for bacteria and virus was similar as well, which is comparable to that in the previous studies (Li et al., 2022; Sun W. et al., 2024). As the development of tNGS products was targeting specific infectious pathogens, our BALF tNGS panel mainly focuses on lower respiratory tract infection pathogens. For bacteria, due to the broader coverage of mNGS, the detection rate of mNGS is higher than that of tNGS in our study. mNGS detected a higher number of *E. faecium*, *C. striatum*, *R. mannitolilytica*, and *B. ambifaria* compared to tNGS, whereas tNGS identified more *S. anginosus* group. *E. faecium* and *C. striatum* are the components of the normal flora of skin and mucous membranes and are often regarded as opportunistic pathogens, particularly in individuals with compromised immune function. In such cases, they may lead to severe infections, thereby possessing significant clinical implications. Although the significance of *R. mannitolilytica* and *B. ambifaria* in lower respiratory tract infections is limited, a rare case of spheroid pneumonia caused by *R. mannitolilytica* was diagnosed through mNGS as reported (Ma et al., 2023). For viruses, tNGS has a greater advantage than mNGS in the detection of RNA viruses, such as SARS-CoV-2, Rhinovirus A, human respiratory syncytial virus, human coronavirus HKU1, and OC43, which could not be detected by the mNGS DNA process. However, the detection rate of several DNA virus such as CMV was also slightly lower than that of mNGS in our study (mNGS, n = 15; and tNGS, n = 5). The missed detection of CMV by tNGS was mainly due to the filtering of the sequence number that did not meet the reporting standard.

In addition, the ability of tNGS and mNGS to simultaneously identify bacteria, fungi, and viruses was also illustrated in our study. By comparing the results of culture and these two NGS-based technologies, the coinfection rate increased from 7.83% to 47.83% and 56.5% with significant difference for tNGS and mNGS, respectively. However, there was no significant difference observed between mNGS and tNGS, suggesting that the detection capabilities of both methods in cases of mixed infection are largely comparable as well.

As advanced diagnostic tools, mNGS and tNGS often elicit high expectations in clinical practice. However, there are several issues that need attention in the result interpretation. mNGS and tNGS have differences in the presentation of the final sequence number. Since tNGS was based on ultra-multiplex PCR, which specifically increases the number and proportion of nucleic acid sequences of the target pathogen, therefore, the sequence number of tNGS in the final report tends to be higher than that of mNGS. Therefore, the results reporting rules of each pathogen of these two methods need to be combined with clinical practice to avoid false negatives or false positives that may occur in bioinformatics analysis. Accordingly, it is essential to engage in comprehensive communication with clinicians regarding the fact that the sequence counts obtained from tNGS and mNGS should not be directly compared horizontally. Moreover, because NGS-based technologies could not differentiate between colonizing and pathogenic pathogens, it is essential to consider the pathogenic significance when detecting colonizing bacteria with low sequence, such as *Candida albicans*, in BALF.

Our study had several limitations. First, it was a single-center retrospective study, which may limit the generalizability of the results and introduce potential biases related to the specific patient population. Second, the sample size was relatively small, and several cases lacked paired traditional results including G test and IF, owing to the restriction of enrolled cases.

Collectively, in our cohort of 115 patients, tNGS showed comparable diagnostic value to mNGS and was significantly superior to CMT. By focusing on specific pathogen-related gene regions, tNGS not only reduces the volume of sequencing data but also lowers costs and enhances cost-effectiveness for detecting common pathogens. In our lab, tNGS completes the process from sample processing to sequencing in just 15–16 h, compared to mNGS of 20–24 h, facilitating rapid clinical diagnosis. Additionally, for non-critical patients, targeted detection by tNGS can resolve most issues and shows higher sensitivity in detecting low-abundance pathogens, making it highly applicable in clinical settings. Given these practical advantages of tNGS in clinical microbiology, patients with lower respiratory tract infections can be referred for tNGS alongside traditional pathogen detection methods to identify common pathogens. Conversely, mNGS is more appropriate for critical illnesses, particularly in cases of unexplained infections. However, due to the variations in wet and dry experimental procedures across different laboratories, both NGS-based methods currently lack widely accepted standards and quantitative thresholds; therefore, NGS is not intended to supplant traditional diagnostic methods. Instead, NGS-based methodologies should be integrated flexibly with conventional techniques to enhance clinical diagnostic accuracy and inform treatment strategies effectively.

## Data Availability

The datasets presented in this study can be found in online repositories. The names of the repository/repositories and accession number(s) can be found below: https://ngdc.cncb.ac.cn/?lang=zh, PRJCA032778.
