# Supplementary figures and images for: Comparative of metagenomic and targeted next-generation sequencing in lower respiratory tract fungal infections
Source: Front Cell Infect Microbiol. 2025 May 26;15:1534519. doi: 10.3389/fcimb.2025.1534519 (PMC12146372; doi:10.3389/fcimb.2025.1534519)

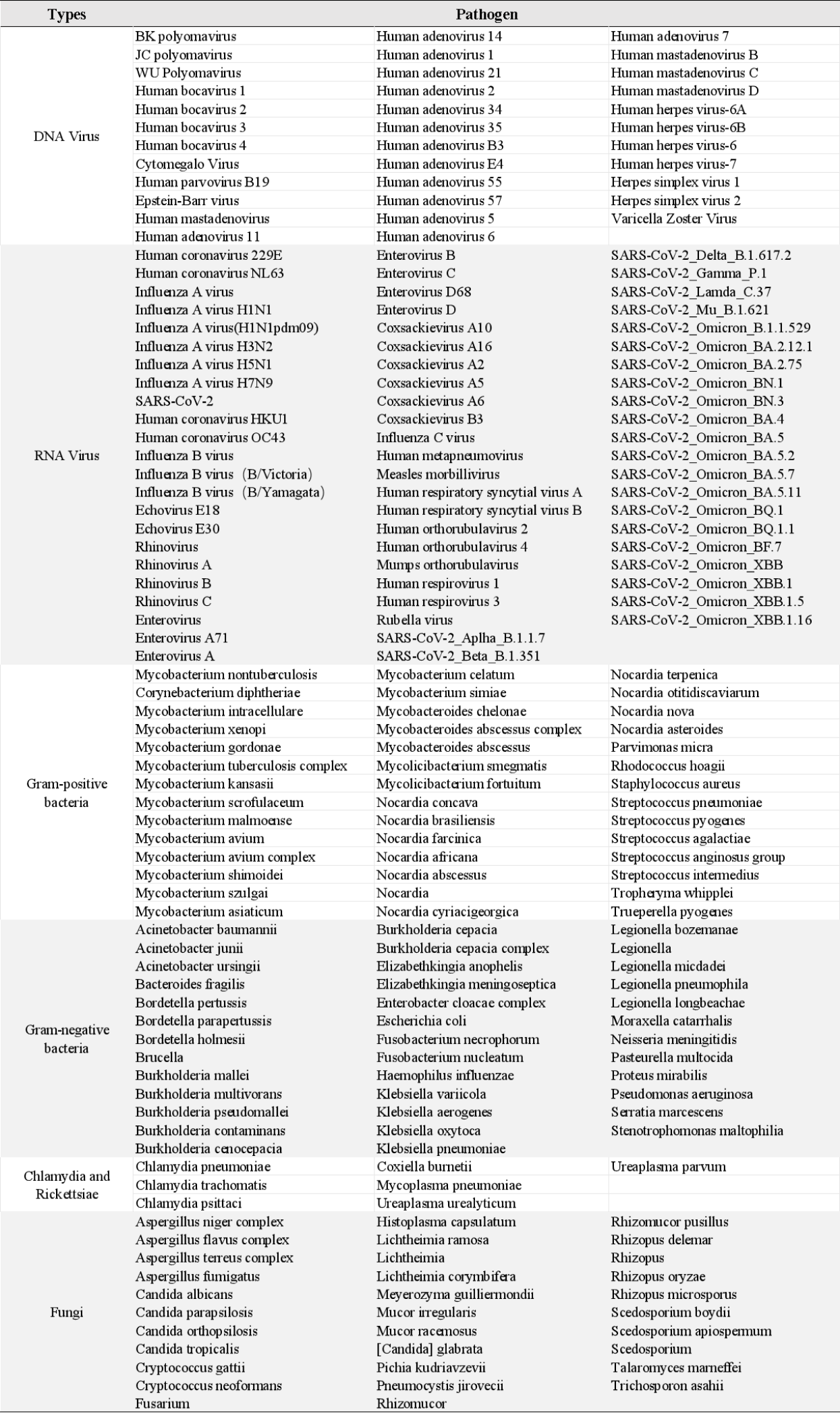

Supplement: Supplementary file 1 [file Image1.tif]
